# Our Mortuary Reports

**Published:** 1880-11

**Authors:** 


OUR MORTUARY REPORTS.—We take this occasion to express our thanks to Mr. A. R. Carter, the indefatigable and gentlemanly clerk of the City Health Commissioner, for his courtesy in compiling the monthly mortuary reports which have appeared in this journal. It has been a labor of love with Mr. Carter, as the work is extra official. His desire to see his native city correctly reported in this particular, has been his only incentive, or expectation of reward.

Mr. Robert Seyboth, of the U. S. Signal Service Office, in this city, has likewise placed us under obligations to him, for his promptness and kindness in furnishing monthly meteorological and thermometrical reports from his office. Our readers can rely upon the correctness of the reports made by both of these gentlemen.

